# Changes of Photosynthetic Behaviors in *Kappaphycus alvarezii* Infected by Epiphyte

**DOI:** 10.1155/2011/658906

**Published:** 2011-08-10

**Authors:** Tong Pang, Jianguo Liu, Qian Liu, Wei Lin

**Affiliations:** ^1^Institute of Oceanology, Chinese Academy of Sciences, Qingdao, Shandong 266071, China; ^2^Graduate University of Chinese Academy of Sciences, Beijing 10049, China

## Abstract

Epiphytic filamentous algae (EFA) were noted as a serious problem to reduce the production and quality of *K. alvarezii*. The morphological studies revealed that the main epiphyte on *K. alvarezii* was *Neosiphonia savatieri* in China. Though the harmful effects of EFA on the production of *K. alvarezii* have been reported, the detailed mechanism of the *N. savatieri* in limiting the production of *K. alvarezii* has not been studied yet. The present paper studied the effects of *N. savatieri* infection on photosynthetic behaviors in *K. alvarezii* by detecting chlorophyll fluorescence transient in vivo. The results revealed that damage of oxygen-evolving complex (OEC), decrease of active reaction centers (RCs), and the plastoquinone (PQ) pool as well as significant reduction in the performance indexes (PI) of PSII were caused by the infection of *N. savatieri*. The influence of *N. savatieri* on photosynthetic activity of *K. alvarezii* should be one of the important reasons to reduce the production of *K. alvarezii* infected by *N. savatieri*.

## 1. Introduction

*Kappaphycus alvarezii *(Solieriaceae, Rhodophyta) have been farmed as raw materials for carrageenan production in many countries since 1970s [1]. However, the carrageenan industry was faced with raw material problems relating to quality and quantity [2]. Epiphyte infection was one of the main reasons causing the decrease of quality and quantity of raw materials. 

Epiphytic filamentous algae (EFA) were noted as a serious problem since early *K*.* alvarezii* cultivation [3]. The outbreaks of EFA (*Polysiphonia* sp., *Neosiphonia savatieri*) in Philippines and Malaysia, which caused a decrease of *K*.* alvarezii* production, were reported by Hurtado et al. [4] and Vairappan [5], respectively. Vairappan [5] noted that the outbreak of EFA correlated with drastic changes in seawater temperature and salinity from March to June and September to November. For further information, the infected *K*.* alvarezii* from carrageenophyte farms in the Philippines, Indonesia, Malaysia, and Tanzania were collected and studied to establish baseline information on the epiphyte's identity, density, symptoms, and secondary infection on the host seaweed [6]. Vairappan et al. [6] found out that the dominant epiphyte in these four culture areas was *N*. *apiculata*.

EFA comprise numerous species of filamentous algae that attach to the cortical layer of the host thalli. They leave the* K*.* alvarezii* stunted, rough, and poorly branched [7]. However, more detailed physiological mechanism of EFA on *K*.* alvarezii* has not been thoroughly studied yet. Anyway, EFA cannot do harm to host thalli without two channels: exchange of materials and energy metabolism. Photosynthesis is the basic anabolism and the only way light energy transfers into electric then chemical energy in plants. Thus, the process is vital for algal growth and survival. Chlorophyll a (Chl a) fluorescence analysis has been proved to be a very useful, noninvasive tool for plant study and more specifically the behavior of photosystem II [8–11]. Recent improvements in detecting the fluorescence signal through direct and time-resolved measurements could provide detailed information on the fast fluorescence rise. All oxygenic photosynthetic materials investigated so far show a polyphasic rise consisting of the basic steps from the “origin” (*O*) through two “inflections” (*I*_1_, designated as J, and *I*_2_, termed I) to a “peak” fluorescence [12]. The O-J-I-P polyphasic transient was found to change its shape according to changes in the environment conditions [11, 13, 14]. The analysis of the fast fluorescence rise according to the JIP test allows the derivation of several expressions leading to the actual description of a photosynthetic sample in a current physiological state [8]. Here, we presented the dominant EFA in China and its impacts on photosynthetic behaviors in *K*. *alvarezii* by using continuously Chl a fluorescence, which were recorded in vivo with high time resolution and analyzed according to JIP-test.

## 2. Materials and Methods

### 2.1. Materials

Both infected and healthy green *K*.* alvarezii* were collected from Lian Bay, Hainan province, China (18°27′N, 110°5′E). Detritus on the materials were cleaned by seawater. Sections and dominant epiphytes was removed by a razor blade and then transferred to microscope slides. Slides were viewed at 100x magnification under optical microscope. Images were taken using an attached Cannon digital camera to investigate the morphological characters of epiphyte.

EFA-infected green* K*.* alvarezii* were precleaned with a soft brush to remove all the epiphytes and contaminants and then were brought to our laboratory beside the bay accompanied with the healthy ones to carry out the physiological studies.

### 2.2. Chl a Fluorescence Measurement

Algal thalli, about 3-4 mm in diameter and 3 cm in length, were selected, respectively, from infected and healthy green *K*.* alvarezii*. Each thallus was transferred into one capped transparent glass vial filled with seawater, and subsequently the vial was incubated at room temperature in darkness for 15 min. Chl a fluorescence of dark-adapted sample was measured by a plant efficiency analyzer (Handy PEA, Hansatech UK) and a single vial adapter for liquid-phase samples (HPEA/LPA2 Hansatech, UK). Red light of 650 nm wavelength (1500 *μ*mol m^−2^ s^−1^) was continuously provided for 1 s. The fluorescence transients were recorded in a time span from 10 *μ*s to 1 s. For the first 300 *μ*s, fluorescence was sampled at 10 *μ*s intervals. The time resolution of digitization was then switched to slower acquisition rates as the kinetics of the fluorescence signal slow. Each group of experiments was done for four times.

### 2.3. Analysis of OJIP Chl a Fluorescence Induction Transient

Each transient was analyzed according to JIP-test [15–18] by utilizing the following data: the minimal fluorescence intensity (*F*_*O*_) when all RCs are open, the maximal fluorescence intensity (*F*_*m*_), assuming that excitation intensity is high enough to close all the RCs of PSII, and the fluorescence intensities at times 300 *μ*s (*F*_*K*_), 2 ms (*F*_*J*_), and 30 ms (*F*_*I*_). Based on the above data, the following parameters were then calculated: the relative variable fluorescence intensity at the *J*-step, *V*_*J*_ ≡ (*F*_*J*_ − *F*_*O*_)/(*F*_*m*_ − *F*_*O*_); the relative variable fluorescence intensity at the *K*-step, *V*_*K*_ ≡ (*F*_*K*_ − *F*_*O*_)/(*F*_*m*_ − *F*_*O*_); the approximated initial slope of the fluorescence transient, *M*_*O*_ ≡ 4(*F*_*K*_ − *F*_*O*_)/(*F*_*m*_ − *F*_*O*_); the total complementary area above the O-J-I-P transient, Area = ∫_0_^*tF*_*m*_^(*F*_*m*_ − *F*_*t*_)*dt*.

The normalized total complementary area above the O-J-I-P transient (reflecting single-turnover *Q*_*A*_ reduction events) is *S*_*m*_ ≡ (Area)/(*F*_*m*_ − *F*_*O*_); the times of *Q*_*A*_ have been reduced to *Q*_*A*_^−^ in the time span from *t*_0_ to *t*_*F*_*m*__, *N* ≡ *S*_*m*_ × *M*_*O*_ × (1/*V*_*J*_).

The maximum quantum yield of primary photochemistry is*φ*_*P*_*O*__ ≡ TR_*O*_/ABS = [1 − (*F*_*O*_/*F*_*m*_)]; the probability that a trapped exciton moves an electron into the electron transport chain beyond *Q*_A_^−^ is *ψ*_*O*_ ≡ ET_*O*_/TR_*O*_ = (1 − *V*_*J*_); the quantum yield for electron transport is*φ*_*E*_*O*__ ≡ ET_*O*_/ABS = [1 − (*F*_*O*_/*F*_*m*_)] × *ψ*_*O*_.

The specific energy fluxes (per *Q*_A_-reducing PSII reaction center (RC)) for the energy absorbed isABS/RC = *M*_*O*_ × (1/*V*_*J*_)×(1/*φ*_*P*_*O*__); the energy trapped isTR_*O*_/RC = *M*_*O*_ × (1/*V*_*J*_); the electron transported isET_*O*_/RC = *M*_*O*_ × (1/*V*_*J*_) × *ψ*_*O*_; and the energy dissipated isDI_*O*_/RC = (ABS/RC)−(TR_*O*_/RC).

Phenomenological energy fluxes (per excited cross-section (CS)) for absorption (ABS/CS), trapping (TR*_O_*/CS), electron transport (ET*_O_*/CS), and dissipation (DI*_O_*/CS) were calculated by the following equations: ABS/CS_*O*_ ≈ *F*_*O*_ (at *t* = *t*_0_); ABS/CS_*m*_ ≈ *F*_*m*_ (at *t* = *t*_*F*_*m*__); TR_*O*_/CS_*O*_ = *φ*_*P*_*O*__ × (ABS/CS_*O*_) (at *t* = *t*_0_); TR_*O*_/CS_*m*_ = *φ*_*P*_*O*__ × (ABS/CS_*m*_) (at *t* = *t*_*F*_*m*__); ET_*O*_/CS_*O*_ = *φ*_*E*_*O*__ × (ABS/CS_*O*_) (at *t* = *t*_0_); ET_*O*_/CS_*m*_ = *φ*_*E*_*O*__ × (ABS/CS_*m*_) (at *t* = *t*_*F*_*m*__); DI_*O*_/CS_*O*_ = (ABS/CS_*O*_)−(TR_*O*_/CS_*O*_) (at *t* = 0); DI_*O*_/CS_*m*_ = (ABS/CS_*m*_)−(TR_*O*_/CS_*m*_) (at *t* = *t*_*F*_*m*__).

The density of reaction centers per exited cross-section was computed by the equations below: RC/CS_*O*_ = *φ*_*P*_*O*__ × (*V*_*J*_/*M*_*O*_)×(ABS/CS_*O*_) (at *t* = 0); RC/CS_*m*_ = *φ*_*P*_*O*__ × (*V*_*J*_/*M*_*O*_)×(ABS/CS_*m*_) (at *t* = *t*_*F*_*m*__).

The performance indexes for absorption (PI_ABS_) and per excited cross-section (PI_CS_) were calculated as follows: PI_ABS_ ≡ (RC/ABS)×[*φ*_*P*_*O*__/(1 − *φ*_*P*_*O*__)]×[*ψ*_*O*_/(1 − *ψ*_*O*_)]; PI_CS_*O*__ ≡ (RC/CS_*O*_)×[*φ*_*P*_*O*__/(1 − *φ*_*P*_*O*__)]×[*ψ*_*O*_/(1 − *ψ*_*O*_)] (at *t* = *t*_0_); PI_CS_*m*__ ≡ (RC/CS_*m*_)×[*φ*_*P*_*O*__/(1 − *φ*_*P*_*O*__)]×[*ψ*_*O*_/(1 − *ψ*_*O*_)] (at *t* = *t*_*F*_*m*__).

### 2.4. Chlorophyll a Measurement

Cleaned algal thalli, 3-4 mm in diameter and 0.5 g fresh weight, were selected, respectively, from infected and healthy green *K*.* alvarezii*. The thalli were homogenized in 5 mL of 95% ethanol for 15 min then were centrifuged at 1000 rpm for 5 min. After centrifugation, 4 mL supernatant was transferred into a colorimetric tube and diluted to 25 mL with 95% ethanol. Absorbance was measured by 722 s spectrophotometer (Shanghai precision & scientific instrument CO., LTD) at 665 nm and 649 nm. Each group of experiments was done for 3 times. Pigment concentration was calculated according to Wintermans and de Mots [19],

(1) $$\begin{array}{cc} & \text{Chl}\;\text{a}\;\left(\mu \text{g}/\text{g}\right)\\ & \;=\left(13.7{\text{OD}}_{665\;\text{nm}}-5.76{\text{OD}}_{649\;\text{nm}}\right)\times \frac{\text{dilution}\;\text{rate}}{0.5\;\text{g}}. \end{array}\;$$

### 2.5. Phycobiliprotein Measurement

Cleaned algal thalli, 3-4 mm in diameter and 0.5 g fresh weight, were selected, respectively, from infected and healthy green *K*.* alvarezii*. The thalli were chopped into 3 mm^3^ and then homogenized in 3 mL of 10 mM CaCl_2_ solution, which was stocked in 4°C for 12 hours prior to the experiment, for 15 min. Subsequently, the homogenized solution was transferred into a colorimetric tube then diluted to 25 mL with 10 mM CaCl_2_. After that, the colorimetric tubes were incubated at 4°C in dark for 48 hours. Absorbance of the supernatant at 562 nm, 615 nm, and 652 nm was measured by 722 s spectrophotometer. Each group of experiments was done for 3 times. Phycobiliproteins were calculated according to Venkataraman [20] as below:

(2) $$\begin{array}{cc} & \text{Phycocyanin}\;\left(\text{PC}\right)\left(\text{mg}/\text{g}\right)\\ & \;=\frac{\left({\text{OD}}_{615\;\text{nm}}-0.474{\text{OD}}_{652\;\text{nm}}\right)}{5.34}\times \frac{\text{dilution}\;\text{rate}}{0.5\;\text{g}}, \end{array}$$

(3) $$\begin{array}{cc} & \text{Phycoerythrin}\;\left(\text{PE}\right)\left(\text{mg}/\text{g}\right)\\ & \;=\frac{\left({\text{OD}}_{562\;\text{nm}}-2.41\text{PC}-0.849\text{APC}\right)}{9.62}\times \frac{\text{dilution}\;\text{rate}}{0.5\;\text{g}}, \end{array}$$

(4) $$\begin{array}{cc} & \text{Allophycocyanin}\;\left(\text{APC}\right)\left(\text{mg}/\text{g}\right)\\ & \;=\frac{\left({\text{OD}}_{562\;\text{nm}}-0.208{\text{OD}}_{615\;\text{nm}}\right)}{5.09}\times \frac{\text{dilution}\;\text{rate}}{0.5\;\text{g}}, \end{array}$$

(5) $$\begin{array}{cc} & \text{Phycobiliprotein}\;\left(\text{PEP}\right)\left(\text{mg}/\text{g}\right)=\text{PC}+\text{APC}+\text{PE}. \end{array}$$

### 2.6. Statistics

Statistical analyses were performed using SPSS 13.0 software (SPSS Inc., Chicago, USA). Independent sample *t*-test at *P* < 0.05 was used to test the significant differences between the infected and the healthy controls.

## 3. Results

### 3.1. Dominant Epiphytes on the *K. alvarezii*

**Figure 1:**
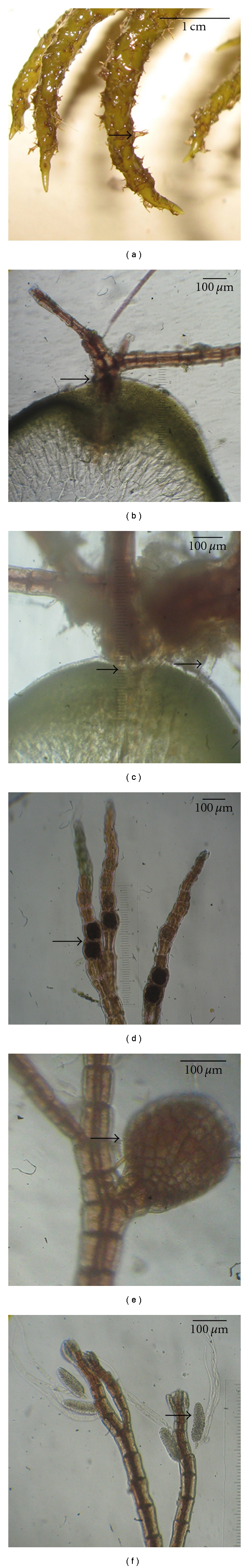
Epiphytes on the *K. alvarezii. *(a) Epiphytes on the surface of *K. alvarezii*. (b) Epiphyte on the transverse section of *K. alvarezii* and a single primary rhizoid penetrate through the outer cortical cells to inner cortical cells. (c) Epiphyte on the transverse section of *K. alvarezii* and a tuft of secondary rhizoids that are cut off from the pericentral cells of lower segments, (d) tetrasporangial branches of epiphyte, (e) broadly ovoid cystocarp of epiphyte, and (f) spermatangial branches of epiphyte.

The dominant epiphytes are brownish red and rigid and have percurrent main axes that reach 2–15 mm. The epiphyte thalli grow on the surface of *K*.* alvarezii* solitarily and close to each other in the peak season (Figure 1(a)). A basal attachment system of the axis is at first composed of a primary rhizoid only (Figure 1(b)), and later forms a tuft of rhizoids by the production of secondary rhizoids that cut off from the pericentral cells of lower segments (Figure 1(c)). The primary rhizoid often penetrates through the outer cortical cells of *K. alvarezii* to medullary layer (Figure 1(b)). The main axes are 60–250 *μ*m in diameter, with segment length 0.5–1.0-fold of diameters. The axes abruptly taper at the apices. Each vegetative segment consists of 4 pericentral cells and lacks cortical cells. The axis produces vegetative trichoblasts or first-order branches from each segment in a spiral manner. Tetrasporangia are formed in the distal segments, one per segment, in a spiral manner. Mature tetrasporangia are 90–110 *μ*m in diameter and protuberant (Figure 1(d)). Procarpial trichoblasts replace vegetative trichoblasts or lateral branches and appear on the distal portion of branches. Each procarpial trichoblast produces a single procarp on the suprabasal segment. The procarp consists of a three-celled carpogonial branch and initials of two sterile groups, one two-celled and lateral, and the other one-celled and basal (Figure 1(e)). Mature cystocarps are broadly ovoid or napiform with 200–350 *μ*m × 200–300 *μ*m in size. Spermatangia are produced on a lateral of fertile trichoblasts that issues from the suprabasal segment. Mature spermatangial branches are conical with 130–200 *μ*m × 45–60 *μ*m in size. They have a one-celled sterile suprabasal segment and the basal segment embedded in the parental branch (Figure 1(f)).

Rhizoids cut off from the pericentral cells of the lower segments, the production of lateral branch in a spiral arrangement, three-celled carpogonial branches, spermatangial trichoblasts with a sterile lateral, and spiralled tetrasporangia found in the epiphyte ally it with *Neosiphonia* than *Polysiphonia *[21]. In addition to these features, the morphology and size of the main axes, tetrasporangia, carpogonial, and spermatangial all ally it with *N*.* savatieri *than *N*.* apiculata *[22, 23]. Therefore, based on the results above the dominant EFA in Lian Bay, Hainan province, China are *N*.* savatieri*.

### 3.2. Fast Chl a Fluorescence Kinetics, O-JIP

**Figure 2:**
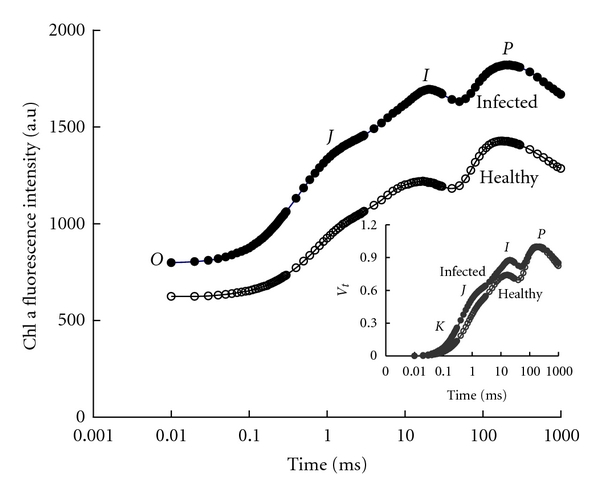
Changes of the fluorescence kinetics, O-J-I-P, plotted on logarithmic time scale from 10 *μ*s to 1 s of *N*.* savatieri*-infected *K*.* alvarezii* (the original data without any normalization). In the insert chart, a relative variable fluorescence, *V*_*t*_ = (*F*_*t*_ − *F*_*O*_)/(*F*_*m*_ − *F*_*O*_) from 10 *μ*s to 1 s, is shown. Values present mean of four replicates.

Figure 2 showed the fast Chl a fluorescence induction kinetics of both the healthy and the infected* K*.* alvarezii*. When the thalli of *K*.* alvarezii* are exposed to saturating actinic light, the Chl a fluorescence curves start from the initial F_O_ intensity and increase to a peak (*P* or *F*_*m*_). When the curves were plotted on logarithmic scale, two intermediate steps *F*_*J*_ (about 2 ms) and *F*_*I*_ (about 30 ms) can be found between *F*_*O*_ and *F*_*m*_. To visualize the comparative effects of *N*.* savatieri* infection on each step, the curves were replotted as relative variable fluorescence, *V*_*t*_ = (*F*_*t*_ − *F*_*O*_)/(*F*_*m*_ − *F*_*O*_) in the insert chart of Figure 2. Based on the insert chart in Figure 2, certain increases in the peaks at *K*-, *J*-, and *I*-steps were found in the *N*.* savatieri*-infected *K*.* alvarezii *compared with the healthy seaweed.

### 3.3. Donor and Acceptor Side of PSII Reaction Center

Increase amplitude in *K*-step was used as a specific indicator of damage to the oxygen-evolving complex (OEC) [12, 17, 24, 25]. The amplitude in the *K*-step of *K*. *alvarezii*, expressed as the ratio *V*_*K*_, was shown in Table 1. An obvious increase in *V*_*K*_ was observed in *N. savatieri*-infected *K*.* alvarezii*, which reflected that the OEC of host was at least partly damaged. Meanwhile, the number of RCs per excited cross-section (RC/CS_*O*_ or RC/CS_*m*_) was reduced in *K*.* alvarezii* after *N. savatieri* infection. *V*_*J*_ was used as an indicator of the proportion of active reaction centers (RCs) [12, 15, 17]. The increase of *V*_*J*_ (Table 1) further indicated that the number of active RCs in the *N. savatieri*-infected seaweed obviously decreased. 

The approximated initial slope of the fluorescence transient (*M*_*O*_), a profile of the maximal reduction rate of *Q*_*A*_, increased by 89.5% in *N. savatieri-*infected *K*.* alvarezii *(Table 1). However, the normalized total complementary area above the O-J-I-P transient (*S*_*m*_), the energy needed to reduce all the *Q*_*A*_, decreased by 29.5% (Table 1). The increase in *M*_*O*_ and decrease in *S*_*m*_ was one indicator of the decrease in the plastoquinone (PQ) pool [12, 15, 17, 25]. Therefore, the changes of the *M*_*O*_ and *S*_*m*_ in *K*.* alvarezzi,* after *N. savatieri* infection showed the plastoquinone (PQ) pool of the host decreased. What is more, *N* ≡ *S*_*m*_ × *M*_*O*_ × (1/*V*_*J*_), the negligible change (2.7%) in the turnover number of *Q*_*A*_(*N*) were induced by the integrated effects of changes in *M*_*O*_, *V*_*J*_, and *S*_*m*_.

### 3.4. Energy Distribution via PSII Reaction Center

After *N. savatieri* infection, the energy fluxes via PSII reaction centers (RCs) in *K*.* alvarezii* significantly changed. The light energy for absorption (ABS/RC) and trapping (TR_*O*_/RC) in *N. savatieri* infected *K*.* alvarezii*-increased by 49.5% and 50% (Table 2). However, the specific energy fluxes (per *Q*_*A*_-reducing PSII reaction center (RC)) for the energy dissipated (DI_*O*_/RC) increased significantly (Table 2), and the energy for electron transported per reaction center (ET_*O*_/RC) in the *N. savatieri*-infected *K*.* alvarezii* did not change so significantly. Therefore, most of the energy trapped was not used for photosynthesis but dissipated by the reaction centers.

Similarly, the energy distribution was further expressed via excited cross-section. Regardless of Chl a fluorescence at *t*_*F*_*O*__ or *t*_*F*_*m*__, the phenomenological energy fluxes per excited cross section (CS) for absorption (ABS/CS), trapping (TR_*O*_/CS), and dissipation (DI_*O*_/CS) in *K*.* alvarezii *increased by 27% (Table 3) after *N. savatieri* infection. The increase in the DI_*O*_/CS acted as a counterbalance to the increase of TR_*O*_/CS. Therefore, the change of electron transport per excited cross section (ET_*O*_/CS) in *K*.* alvarezzii *after *N. savatieri* infection was negligible (Table 3).

### 3.5. Performance Indexes (PI) and Quantum Yields

The probability that trapped exciton moves an electron into the electron transport chain beyond *Q*_A_^−^(*ψ*_*O*_), and the quantum yield for electron transport (*φ*_*E*_*O*__) decreased by 23.5% and 24.1% in *N. savatieri*-infected *K*.* alvarezii* comparing with the healthy control (Table 4). No significant changes in the maximum quantum yield of primary photochemistry (*φ*_*P*_*O*__) were found in the infected *K*.* alvarezii* compared with the control. However, the comprehensive performance indexes (PI) significantly decreased (Table 4). The average PI_ABS_,  PI_CS_*O*__, and PI_CS_*m*__ in *N. savatieri*-infected *K*.* alvarezii* decreased by 57.7%, 44%, and 42.9%, respectively, compared with the healthy control (Table 4). 

### 3.6. Photosynthetic Pigments

Chl a and phycobiliprotein content in *K*.* alvarezii* changed significantly (Table 5) after the seaweed was infected by *N. savatieri* (*P* < 0.05). The content of Chl a, phycocyanin (PC), phycoerythrin (PE), allophycocyanin (APC), and phycobiliprotein (PBP) in *N. savatieri*-infected* K*.* alvarezii* increased about 56.4%, 104.5%, 146.2%, 139.4%, and 130.9% compared with the healthy control, respectively (Table 5). The pigments increase in *N. savatieri*-infected *K*. *alvarezii* (Table 5) was much higher than the increase of ABS/CS and TR_*O*_/CS (Table 4). The above results indicated a relative decrease in the light energy absorbed per pigment. 

## 4. Discussion

The changes in PSII performance of the photosynthetic apparatus caused by environmental stress or senescence have been explored widely by applying the chlorophyll fluorescence technique in higher plants [8, 26–31]. However, there is not detailed knowledge on the influence of epiphyte on the photosynthetic activity of its host. In the present study, we have demonstrated the response of PSII of *K*.* alvarezii* to *N*.* savatieri* infection. The Chl a fluorescence transient recorded with high time resolution was analyzed by the JIP-test in order to quantify the PSII behaviors in *K*.* alvarezii *after *N. savatieri* infection. 

*φ*_*P*_*O*__ changed slightly; however, PI decreased significantly in *N. savatieri*-infected *K*.* alvarezii*. The PI was calculated from three components, which depend on the reaction center density, the trapping efficiency, and the electron transport efficiency. The above changes of PI showed that photosynthesis in the infected *K*.* alvarezii* was inhibited which could partly explain the phenomenon of stunted, rough, and poorly branched carrageenan producing seaweed arisen by epiphyte infection [7]. Moreover, our results proved that PI is more sensitive to environmental change than *φ*_*P*_*O*__ and correlates well with plant vigor and performance again that agrees with the research by Hermans et al. [32].

Chlorophyll and phycobiliprotein content in *N. savatieri*-infected *K*.* alvarezii* was increased to 156% and 230% (Table 5). Therefore, the energy fluxes for absorption and trapping in *N. savatieri*-infected *K*.* alvarezii* were increased (Tables 2 and 3). However, the negligible changes of ET_*O*_/CS (Table 3) and *φ*_*P*_*O*__ (Table 4) indicated that the trapped energy was not efficiently used for electron transport. The damage of OEC, decrease in RC number, and reduction of PQ pool could further explain why the light trapped in *N. savatieri*-infected* K*.* alvarezii* was not sufficiently consumed timely for photosynthesis.

The side impacts of epiphyte on *K*.* alvarezii* growth were not only limited to photochemical reactions. Largo et al. [33] reported that light intensity of less than 50 *μ*moL photon m^−2^ could induce the decay of* K*.* alvarezii*. *N. savatieri* occupied the outsurface of *K*. *alvarezii* and shielded the host from getting enough light. Moreover, both *N*.* savatieri* and *K*.* alvarezii* all belonged to Rhodophyta species and owned similar types of photosynthetic pigments that aggravated the competition of light absorption between them. The competition between *N*.* savatieri* and *K*.* alvarezii* seriously decreased the ambient light. However, the infected *K*.* alvarezii* tried to acclimate itself to the low-light conditions by increasing its photosynthetic pigments, especially phycobiliprotein (Table 5). Unfortunately, the adaptive regulation seemed to be meaningless for EFA-infected *K*. *alvarezii* because of the decrease in active RC number, damage of OEC, and reduction of PQ pool as mentioned above. Glenn and Doty [34] reported that culture of *K*.* alvarezii* required high levels of water motion provided by strong and consistent trade winds. *N*.* savatieri*, covered on the surface of *K*.* alvarezii*, were bound to reduce the water motion nearby the *K*.* alvarezii* as well as the materials exchange between* K*.* alvarezii *and external environment. Therefore, the production of oxygen by the photosynthesis of *N*.* savatieri* and *K*.* alvarezii *was easy to cause the surplus of oxygen during the daytime. Moreover, the consumption of oxygen by the respiration of *N*.* savatieri* and *K*.* alvarezii* was easy to cause the insufficiency of oxygen during the nighttime. In addition, the epiphyte *N*.* savatieri* competed with host *K*.* alvarezii* for absorbing nutrients (N, P, CO_2_, and other mineral elements). Most of the nutrients dissolving in the water body were first filtered by *N*.* savatieri* before reaching to *K*.* alvarezii*, and so nutrient deficiency inevitably occurred in *K*.* alvarezii* after EFA infection. Dense *N. savatieri* were severe stress for the metabolism of *K*.* alvarezii* by shading, high O_2_ concentrations in the light, anoxic conditions in the dark, and competition of nutrients. Therefore, heavy decay in *K*.* alvarezii* was usually found when the seaweed was infected by* N. savatieri*. 

In conclusion, the dominant EFA on *K*.* alvarezii* in Lian Bay, Hainan province were *N*.* savatieri*. Damage of OECs, decrease of active RCs and the PQ pool and significant reduction in the performance indexes (PI) of PSII were caused by the infection of *N. savatieri* although the seaweed acclimated itself to the low-light condition by increasing its photosynthetic pigments to absorb more light energy. The influence of *N. savatieri* on photosynthetic activity of *K. alvarezii* was one of the important reasons to reduce the production of *K. alvarezii.*

## Figures and Tables

**Table 1:** Profiles reflecting the donor and acceptor side of PSII in the healthy and infected *K*. *alvarezii. *

	*V*_K_*	RC/CS_*O*_	RC/CS_*m*_	*V*_J_*	*M*_O_*	*S*_m_*	*N*
Healthy	0.14 ± 0.03	299 ± 61	695 ± 150	0.49 ± 0.08	0.57 ± 0.11	23.73 ± 2.29	27.52 ± 2.08
Infected	0.27 ± 0.06	259 ± 30	606 ± 70	0.61 ± 0.07	1.08 ± 0.26	16.73 ± 4.54	28.25 ± 3.59
RV	1.929	0.866	0.872	1.245	1.895	0.705	1.027

Values present mean ± SE of four replicates, *indicates significant differences at *P* < 0.05 between the healthy and infected* K*. *alvarezii*, and RV indicates the relative value of infected sample to the healthy sample.

**Table 2:** Profiles reflecting energy flux per reaction center in the healthy and infected *K*.* alvarezii. *

	ABS/RC*	TR_*O*_/RC*	DI_*O*_/RC*	ET_*O*_/RC
Healthy	2.04 ± 0.08	1.16 ± 0.05	0.88 ± 0.04	0.59 ± 0.07
Infected	3.05 ± 0.50	1.74 ± 0.29	1.30 ± 0.24	0.66 ± 0.13
RV	1.495	1.500	1.477	1.119

Values present mean ± SE of four replicates, *indicates significant differences at *P* < 0.05 between the healthy and infected* K*. *alvarezii*, and RV indicates the relative value of infected sample to the healthy sample.

**Table 3:** Profiles reflecting energy flux per excited cross section in the healthy and infected *K*.* alvarezii. *

		ABS/CS	TR_*O*_/CS	DI_*O*_/CS	ET_*O*_/CS
Heathy	*t* = 0	614 ± 147	349 ± 86	264 ± 61	174 ± 17
Infected	*t* = 0	778 ± 68	445 ± 33	333 ± 41	170 ± 27
RV	*t* = 0	1.267	1.275	1.261	0.977
Healthy	*t* = *t*_*F*_*m*__	1427 ± 354	812 ± 207	614 ± 147	405 ± 44
Infected	*t* = *t*_*F*_*m*__	1821 ± 140	1042 ± 94	778 ± 68	401 ± 84
RV	*t* = *t*_*F*_*m*__	1.276	1.283	1.267	0.999

Values present mean ± SE of four replicates and RV indicates the relative value of infected sample to the healthy sample.

**Table 4:** Performance indexes (PI) and quantum yields in the healthy and epiphyte-infected *K*.* alvarezii. *

	*φ*_*P*_*O*__	*ψ*_*O*_	*φ*_*E*_*O*__	PI_ABS_*	PI_CS_O__*	PI_CS_m__*
Healthy	0.57 ± 0.01	0.51 ± 0.08	0.29 ± 0.04	0.71 ± 0.23	411 ± 43	952 ± 97
Infected	0.57 ± 0.02	0.39 ± 0.07	0.22 ± 0.05	0.30 ± 0.15	230 ± 95	544 ± 237
RV	1	0.765	0.759	0.423	0.56	0.571

Values present mean ± SE of four replicates, *indicates significant differences at *P* < 0.05 between the healthy and infected* K*. *alvarezii*, and RV indicates the relative value of infected sample to the healthy sample.

**Table 5:** Effects of *N. savatieri* on the photosynthetic pigments of *K. alvarezii. *

	Chl a(*μ*g/g)*	PC(mg/g)*	PE(mg/g)*	APC(mg/g)*	PBP(mg/g)*
Healthy	45.6 ± 6.8	0.22 ± 0.01	0.13 ± 0.01	0.33 ± 0.02	0.68 ± 0.04
Infected	71.3 ± 1.9	0.45 ± 0.03	0.32 ± 0.03	0.79 ± 0.07	1.57 ± 0.13
RV	1.564	2.045	2.462	2.394	2.309

Values present mean ± SE of four replicates, *indicates significant differences at *P* < 0.05 between the healthy and infected* K*. *alvarezii*, RV indicates the relative value of infected sample to the healthy sample.
